# DDT and Breast Cancer

**DOI:** 10.1289/ehp.11025

**Published:** 2008-04

**Authors:** Robert E. Tarone

**Affiliations:** International Epidemiology Institute, Rockville, Maryland, E-mail: bob@iei.ws

In a recent article, Cohn et al. (2007) noted an association between increased breast cancer risk and *p,p*′-DDT [1,1,1-trichloro-2,2-bis(*p*-chlorophenyl)ethane] exposure early in life. Their article should be interpreted with caution, particularly the estimated 5-fold increase in risk for women born after 1931 the authors reported without qualification in the “Abstract”; this value was repeated in the news article by Manuel (2007). Cohn et al. (2007) evaluated three DDT congeners—that is, *p,p*′-DDT, *o,p*′-DDT [1,1,1-trichloro-2(*p*-chlorophenyl)-2-(*o*-chlorophenyl)ethane], and *p,p*′-DDE [1,1-dichloro-2,2-bis(*p*-chlorophenyl)ethylene]—by various categories of year of birth, yet they found no significantly increased risk estimates for any of the three DDT congeners in multiple comparisons that were not adjusted for the other DDT-related chemicals either in all women or in women born after 1931. The estimated 5-fold increase in risk for the upper tertile of *p,p*′-DDT serum levels was only observed in subgroup analyses that were both restricted to women born after 1931 and adjusted for serum level of *o,p*′-DDT. The impact of the adjustment for *o,p*′-DDT on the risk estimate for *p,p*′-DDT is remarkable in view of the low *o,p*′-DDT levels observed (35% were below the limit of detection). A significant inverse association between *o,p*′-DDT level and breast cancer risk, which was interpreted by Cohn et al. in terms of length of time since DDT exposure, became stronger after adjustment for *p,p*′-DDT levels; presumably this does not indicate a protective effect of recent DDT exposure.

In view of the absence of evidence for an association between *p,p*′-DDE levels and breast cancer risk (Lopez-Cervantes et al. 2004), it seems unlikely that DDT exposure increases the risk of breast cancer. Nonetheless, if the effect of DDT exposure early in life on breast cancer risk is large [a possibility suggested by Cohn et al. (2007)], then the decreasing birth cohort trend in breast cancer risk that has been observed for U.S. baby boomers is even more remarkable (Chu et al. 1999; Tarone 2006, 2007; Tarone and Chu 2000). Women born after 1945 would have been exposed to DDT for each of the first 13 years of life, with increasing exposure through the late 1960s (Wolff et al. 2005), but the birth cohort risk of breast cancer showed a marked decrease among U.S. women for over two decades after 1945. DDT exposure would join a list of other breast cancer risk factors predicting increasing breast cancer risk in baby boomers (Tarone 2006); yet the birth cohort risk of breast cancer decreased for women born after 1945. That the hypothesized association between DDT exposure and breast cancer risk has received far more attention than the paradoxical decreasing risk of breast cancer that has actually occurred among young U.S. women says much about the priorities and focus of environmental epidemiology.
